# Microbiota from Specific Pathogen-Free Mice Reduces *Campylobacter jejuni* Chicken Colonization

**DOI:** 10.3390/pathogens10111387

**Published:** 2021-10-27

**Authors:** Ayidh Almansour, Ying Fu, Tahrir Alenezi, Mohit Bansal, Bilal Alrubaye, Hong Wang, Xiaolun Sun

**Affiliations:** 1 Center of Excellence for Poultry Science, University of Arkansas, Fayetteville, AR 72701, USA; ama056@uark.edu (A.A.); yingfu@uark.edu (Y.F.); tjalenez@uark.edu (T.A.); mb043@uark.edu (M.B.); bilal.alik8@gmail.com (B.A.); hxw01@uark.edu (H.W.); 2 Cell and Molecular Biology (CEMB), University of Arkansas, Fayetteville, AR 72701, USA

**Keywords:** microbiota transplantation, foodborne pathogen, intestine, bacterial colonization, specific pathogen-free

## Abstract

*Campylobacter jejuni*, a prevalent foodborne bacterial pathogen, is mainly transmitted from poultry with few effective prevention approaches. In this study, we aimed to investigate the role of microbiota on *C. jejuni* chicken colonization. Microbiota from specific pathogen-free (SPF) mouse stools were collected as SPF-Aerobe and SPF-Anaerobe. Birds were colonized with SPF-Aerobe or SPF-Anaerobe at day 0 and infected with *C. jejuni* AR101 at day 12. Notably, *C. jejuni* AR101 colonized at 5.3 and 5.6 log_10_ *C. jejuni* CFU/g chicken cecal digesta at days 21 and 28, respectively, while both SPF-Aerobe and SPF-Anaerobe microbiota reduced pathogen colonization. Notably, SPF-Aerobe and SPF-Anaerobe increased cecal phylum *Bacteroidetes* and reduced phylum *Firmicutes* compared to those in the nontransplanted birds. Interestingly, microbiota from noninfected chickens, SPF-Aerobe, or SPF-Anaerobe inhibited AR101 in vitro growth, whereas microbiota from infected birds alone failed to reduce pathogen growth. The bacterium *Enterobacter*102 isolated from infected birds transplanted with SPF-Aerobe inhibited AR101 in vitro growth and reduced pathogen gut colonization in chickens. Together, SPF mouse microbiota was able to colonize chicken gut and reduce *C. jejuni* chicken colonization. The findings may help the development of effective strategies to reduce *C. jejuni* chicken contamination and campylobacteriosis.

## 1. Introduction

*Campylobacter jejuni* colonizes asymptomatically in the intestinal tract of poultry and causes a prevalent foodborne campylobacteriosis around the world [1,2]. *C. jejuni* resistant to macrolides, fluoroquinolones, aminoglycosides, or carbapenems has been detected in samples from children and adults worldwide [1,2,3,4,5,6,7]. More than 20 cases of campylobacteriosis per 100,000 population were reported in the USA in 2019 [8], and more than 220,000 people were affected in Europe in 2019 [9]. The case number was more than the total incidences induced by eight other bacterial pathogens [10]. More than 14.35 cases per 0.1 million people were caused by the pathogen in 2020 [11]. Moreover, *C. jejuni* often causes severe post-infectious complications, such as arthritis [12], the neurodegenerative disorder Guillain–Barré syndrome [13], irritable bowel syndrome [14], and inflammatory bowel diseases (IBD) [15,16].

To reduce campylobacteriosis, different measures have been implemented to reduce enteritis by reducing *C. jejuni* contamination in animal food, particularly pre- and post-harvest poultry. The intervention methods include strict biosecurity on farms [17], vaccines [18], probiotics [19], phages [20], decontamination of poultry carcasses in the post-slaughter process [21], facility design and management, reducing contamination in feed, transportation, and other sources, and other strategies [2]. It is estimated that decreasing *Campylobacter* count on chicken carcasses by 100 times decreases human campylobacteriosis 30-fold [22]. Although those reduction measurements reduce some *C. jejuni* contamination, improved and alternative strategies are much needed, as reflected in the consistent high level of campylobacteriosis incidence reported in the Morbidity and Mortality Weekly Report from the Infectious Disease Database at CDC between 1996 and 2017 [23].

The gastrointestinal tract of humans and animals is inhabited by trillions of microbes, collectively called the microbiota [24,25]. The gut microbiota modulates essential host physiology and various host functions such as the intestinal barrier, nutrition, and immune homeostasis [25,26,27,28,29]. Specific pathogen-free (SPF) *Il10*^−/−^ mice are naturally resistant to *C. jejuni* 81–176-induced colitis, while the mice become susceptible to campylobacteriosis after being treated with the broad-spectrum antibiotic clindamycin [30]. Sequencing and bioinformatic analysis of 16S rDNA revealed that microbiota-mediated bile acid metabolism was essential for preventing *C. jejuni*-induced colitis. Increasing evidence is emerging on gut microbiota preventing *C. jejuni* colonization in poultry [31,32,33]. Apart from naturally transmitting microbiota from wild hens to turkey chicks, the turkey microbiota transmission is disrupted in modern industrialized poultry production, partly because eggs are hatched by a hatchery instead of hens [34]. Poultry chicks obtain their microbiota from the environment and/or farms, where most of the microbes are not natural inhabitants of the bird gut [35]. The application of antibiotics as growth promoters further drives the dysbiosis of birds in commercial poultry production [36]. In our previous studies, we found that transplanting bile acid deoxycholic acid-modulated microbiota to hatched chicks reduced the colonization of *C. jejuni* human clinical isolate 81–176 and chicken isolate AR101 in pre-harvest chickens [37].

Because SPF mice are naturally resistant against a *C. jejuni* infection [30,38], in this study, we hypothesized that SPF mouse microbiota would be able to colonize chickens and reduce *C. jejuni* chicken colonization. Our data indicate that the mouse SPF-Aerobe and SPF-Anaerobe microbiota shaped the chicken intestinal microbiota. Furthermore, the SPF-Aerobe and SPF-Anaerobe indeed reduced *C. jejuni* AR101 in vitro growth and chicken colonization. These findings will help the development of effective strategies against *C. jejuni* chicken colonization.

## 2. Results

### 2.1. Mouse Microbiota Reduced C. jejuni AR101 Chicken Colonization

**Figure 1:**
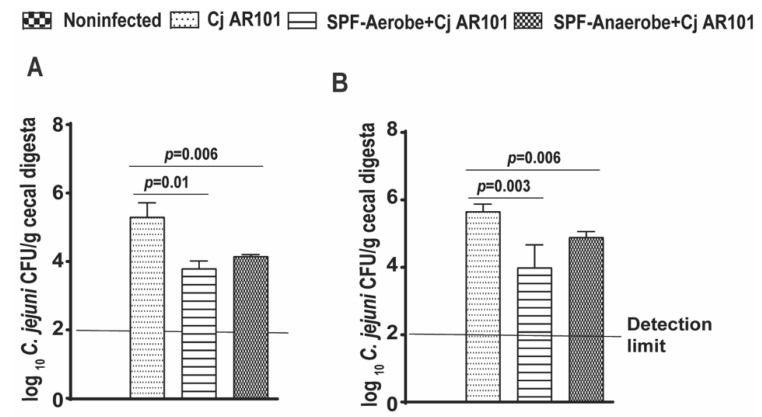
Murine microbiota reduced *C. jejuni* AR101 chicken colonization. Zero-day-old broiler chickens precolonized with SPF-Aerobe and SPF-Anaerobe were infected with *C. jejuni* AR101 at 12 days of age. The birds were euthanized at days 21 and 28. The bird cecal digesta was collected, serially diluted, and cultured on *Campylobacter*-selective agar plates prepared in-house at 42 °C under microaerobic atmosphere. (**A**) *C. jejuni* chicken colonization in the ceca of the birds at day 21. The bird number for each group was: noninfected (n = 10), Cj AR101 (n = 10), SPF-Aerobe (n = 5), and SPF-Anaerobe (n = 10). (**B**) *C. jejuni* chicken colonization in the ceca of the birds at day 28. The bird number for each group was: noninfected (n = 20), Cj AR101 (n = 20), SPF-Aerobe (n = 10), and SPF-Anaerobe (n = 20). All graphs depict the mean + SEM. Significant if *p* < 0.05. The results are representative of three independent experiments.

Mouse SPF-Aerobe and SPF-Anaerobe microbiota was prepared from SPF mouse stools and transplanted to zero-day-old chicks. DNA from *C. jejuni* chicken isolate AR101 was isolated, and 16S rDNA was PCR-amplified, Sanger-sequenced, and confirmed to be in 99.0% alignment with *C. jejuni*. The birds were infected with AR101 at day 12. Consistently with our previous reports [37], *C. jejuni* was not detected in noninfected birds, suggesting clean housing at our poultry facility. Notably, mouse SPF-Aerobe and SPF-Anaerobe microbiota reduced *C. jejuni* AR101 cecal colonization by more than 1-log compared to that of only infected birds (Cj AR101) at day 21 (3.8 ± 0.2 and 4.1 ± 0.0 vs. 5.3 ± 0.4 log_10_ *C. jejuni* CFU/g cecal digesta, respectively) (Figure 1A). The SPF-Aerobe and SPF-Anaerobe continued to reduce *C. jejuni* chicken colonization compared to that of the infected control birds at day 28 (4.0 ± 0.6 and 4.9 ± 0.1 vs. 5.6 ± 0.2 log_10_ *C. jejuni* CFU/g cecal digesta, respectively) (Figure 1B). Notably, the SPF-Anaerobe microbiota with or without *C. jejuni* AR101 infection increased the accumulative body weight compared to that of noninfected birds from day 0 to day 28 (1606 ± 17.7 and 1683 ± 43.1 vs. 1463 ± 47.6 g/bird, respectively) (Figure S1), while SPF-Aerobe microbiota did not increase the bird weight gain. These results suggest that SPF-Aerobe and SPF-Anaerobe effectively reduce *C. jejuni* AR 101 colonization in chickens.

### 2.2. SPF-Aerobe and SPF-Anaerobe Modulated the Chicken Microbiota

**Figure 2:**
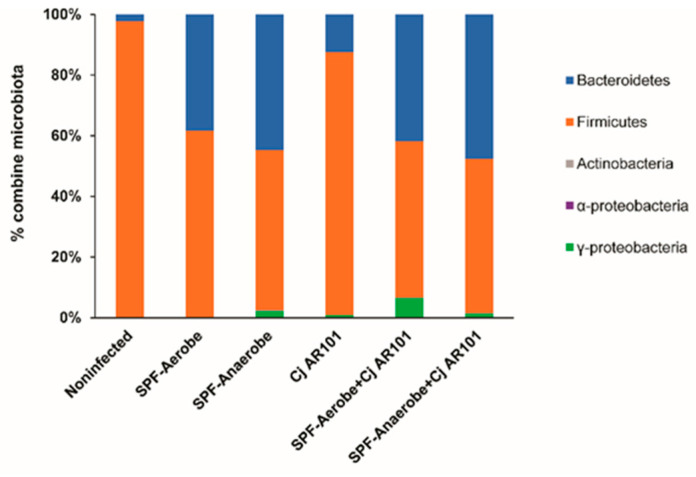
SPF microbiota modified the chicken microbiota at day 28. The birds were colonized with microbiota and infected with *C. jejuni* AR101 at day 12 as in Figure 1. Cecal digesta was collected at day 28, and DNA was extracted. Real-time PCR was performed to calculate bacterial composition at the phylum level. The detailed *p*-values were listed in Table 1. The results are representative of three independent experiments.

We reasoned that the colonization reduction of AR101 in chickens might come from chicken microbiota alteration by the mouse microbiota transplantation. To assess this hypothesis, we used phylum-specific primers to analyze the microbiota composition change. Notably, SPF-Aerobe and SPF-Anaerobe reduced the relative abundance of the phylum *Firmicutes* compared to that of uninfected birds (61.3 and 52.9 vs. 97.5%) and infected birds (51.9 and 50.9 vs. 86.7%, respectively), while the relative abundance of *Bacteroidetes* was increased compared to that of uninfected birds (38.4 and 44.7 vs. 2.3%) and infected birds (42.0 and 47.6 vs. 12.4%, respectively) (Figure 2). Interestingly, *C. jejuni* colonization modulated chicken cecal microbiota of the phyla *Bacteroidetes* and *Firmicutes*. Importantly, most of the relative abundance was significant (Table 1). These results indicate that SPF-Aerobe, SPF-Anaerobe, and *C. jejuni* were able to colonize and change the microbiota in the chicken gut.

### 2.3. Chicken Noninfected Microbiota and Mouse SPF Microbiota Reduced C. jejuni Growth 

**Figure 3:**
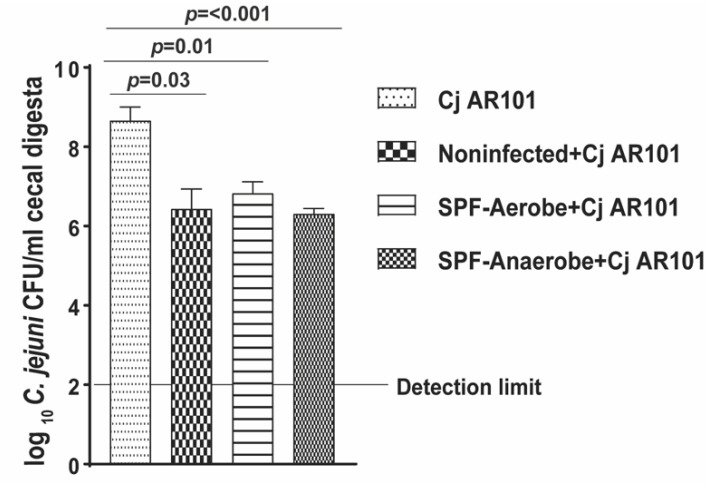
In vitro co-culture of noninfected chicken microbiota and *C. jejuni* AR101. AR101 was co-cultured for 24 h with microbiota from noninfected, SPF-Aerobe, or SPF-Anaerobe birds in vitro. AR101 growth was quantified by serially diluting and plating on the *Campylobacter*-selective agar plates. All graphs depict the mean + SEM. Significant if *p* < 0.05. The results are representative of three independent experiments.

Upon validation of transplanted mouse SPF microbiota reducing *C. jejuni* chicken colonization, we reasoned that the mouse SPF microbiota would directly inhibit *C. jejuni* AR101 growth, while the chicken microbiota would not. To examine this hypothesis, *C. jejuni* AR101 inoculum was co-cultured with microbiota from noninfected, SPF-Aerobe, and SPF-Anaerobe chickens in the Campylobacter Enrichment (CE) Broth at 42 °C for 24 h under anaerobic conditions. Notably, both SPF-Aerobe and SPF-Anaerobe reduced *C. jejuni* AR101 by more than 1-log compared to the *C. jejuni* AR101 culture-alone group (6.8 ± 0.2 and 6.3 ± 0.1 vs. 8.6 ± 0.3 log_10_ *C. jejuni* CFU/mL, respectively) (Figure 3). Interestingly, the microbiota from noninfected chickens also reduced *C. jejuni* AR101 growth by more than 2-log compared to that in the *C. jejuni* AR101 culture alone (6.4 ± 0.4 vs. 8.6 ± 0.3 log_10_ *C. jejuni* CFU/mL, respectively). Notably, each microbiota at 24 h increased the number of CFU compared to that at 0 h (Figure S2).

**Figure 4:**
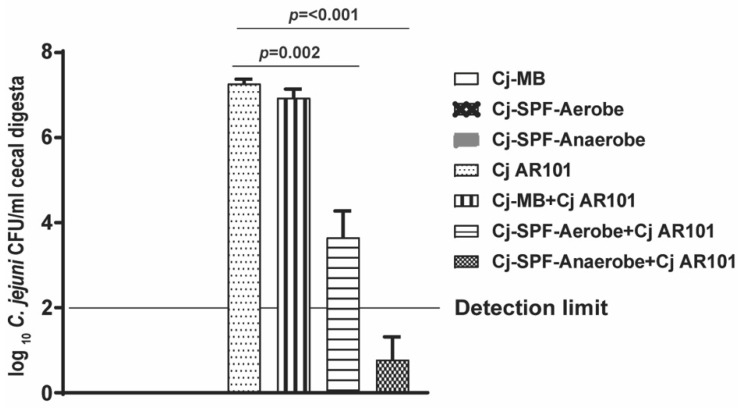
In vitro co-culture of *C. jejuni*-modulated microbiota and *C. jejuni* AR101. *C. jejuni* AR101 was co-cultured with microbiota from infected-alone birds (Cj-MB), transplanted with SPF-Aerobe and infected birds (Cj-SPF-Aerobe), and transplanted with SPF-Anaerobe and infected birds (Cj-SPF-Anaerobe). AR101 growth was quantified by serially diluting and plating on the *Campylobacter*-selective agar plates. All graphs depict the mean + SEM. Significant if *p* < 0.05. The results are representative of three independent experiments.

Because of the unexpected result of noninfected chicken microbiota reducing *C. jejuni* AR 101 in vitro growth, we then modulated our hypothesis that *C. jejuni* possibly modulated chicken microbiota for its growth and colonization. To address this reasoning, we co-cultured *C. jejuni* AR101 with chicken microbiota from only infected birds (Cj-MB), transplanted with SPF-Aerobe and infected birds (Cj-SPF-Aerobe), and transplanted with SPF-Anaerobe and infected birds (Cj-SPF-Anaerobe). Interestingly, the three-chicken microbiota themselves could not grow a single colony on the *Campylobacter*-selective plates (Figure 4), suggesting that *C. jejuni* lost culturability after storing with microbiota. Notably, Cj-MB did not reduce *C. jejuni* AR101 in vitro growth compared to that in positive control of Cj AR101 culture alone. Consistently, Cj-SPF-Aerobe and Cj-SPF-Anaerobe reduced *C. jejuni* AR101 in vitro growth by more than 3-log compared to that in the Cj AR101 culture-alone group (3.7 ± 0.6 and 0.8 ± 0.5 vs. 7.3 ± 0.1 log_10_ *C. jejuni* CFU/mL, respectively). Consistently, each microbiota at 24 h increased number of CFU compared to that at 0 h (Figure S3). These results suggest that *C. jejuni* modulated the chicken microbiota for its growth and colonization, while the transplanted mouse SPF microbiota resisted against pathogen growth. 

### 2.4. An Aerobic Bacterial Isolate Reduced C. jejuni AR101 In Vitro Growth

**Figure 5:**
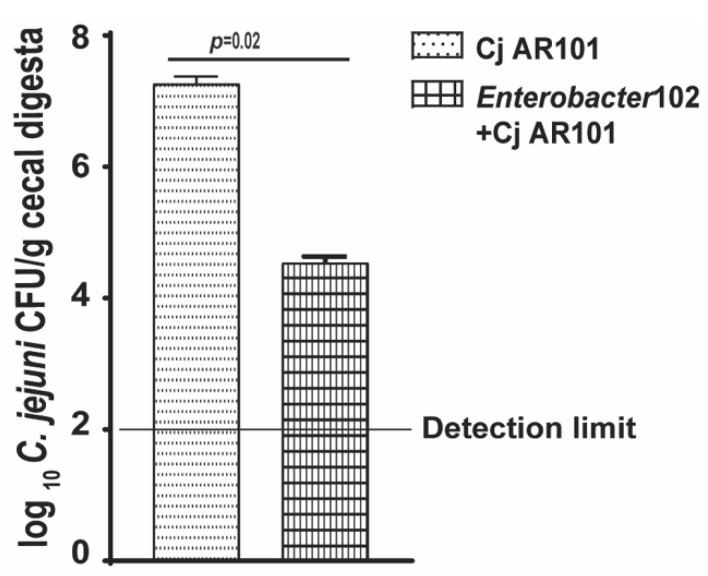
In vitro co-culture of *Enterobacter*102 and *C. jejuni* AR101. *C. jejuni* AR101 was co-cultured with *Enterobacter*102. AR101 growth was quantified by serially diluting and plating on *Campylobacter*-selective agar plates. All graphs depict the mean + SEM. Significant if *p* < 0.05. The results are representative of three independent experiments.

Next, we wanted to identify and isolate the individual bacteria from the protective SPF microbiota. About 100 bacterial colonies were isolated using BHI plates at 42 °C under anaerobic conditions for 48 h. The colonies were individually co-cultured with *C. jejuni* for 24 h, and then *C. jejuni* was enumerated on the *Campylobacter*-selective plates prepared in-house. Unfortunately, none of the bacteria were able to inhibit *C. jejuni* growth using the co-culture method. By accident, during one chicken trial, a bacterial colony from birds gavaged with mouse SPF-Aerobe was able to grow with pink color on the *Campylobacter*-selective plate compared to the dark red color of *C. jejuni*. The bacterial colony was selected and later named *Enterobacter*102. We reasoned that this bacterium might resist *C. jejuni* infection. *Enterobacter*102 was rod-shaped, stained Gram-negative, and had the same size as *E. coli*. *Enterobacter*102 also grew in pink colonies on a MacConkey agar plate. The DNA from *Enterobacter*102 was isolated, and 16S rDNA was PCR-amplified, Sanger-sequenced, and confirmed to be in 95% alignment with *Enterobacter* sp. To functionally dissect the interaction between *Enterobacter*102 and *C. jejuni* AR101, in vitro co-culture was performed. Interestingly, *Enterobacter*102 showed the ability to reduce *C. jejuni* AR101 colonization by more that 2-log compared to that in the Cj AR101 culture-alone group (4.6 ± 0.1 vs. 7.3 ± 0.1 log_10_ *C. jejuni* CFU/mL) (Figure 5). Not surprisingly, the number of *Enterobacter*102 increased at 24 h compared to that at 0 h (Figure S4). Although the reduced *C. jejuni* could result from depleted nutrients in the presence of a microbiota (SPF microbiota or *Enterobacter*102), the comparable growth between Cj AR101 and Cj-MB AR101 in Figure 4 suggested that the microbiota was an important factor influencing *C. jejuni* growth. These results suggested that *Enterobacter*102 has potential to reduce *C. jejuni* AR101 chicken colonization. 

### 2.5. Enterobacter102 Reduced C. jejuni AR101 Chicken Colonization

**Figure 6:**
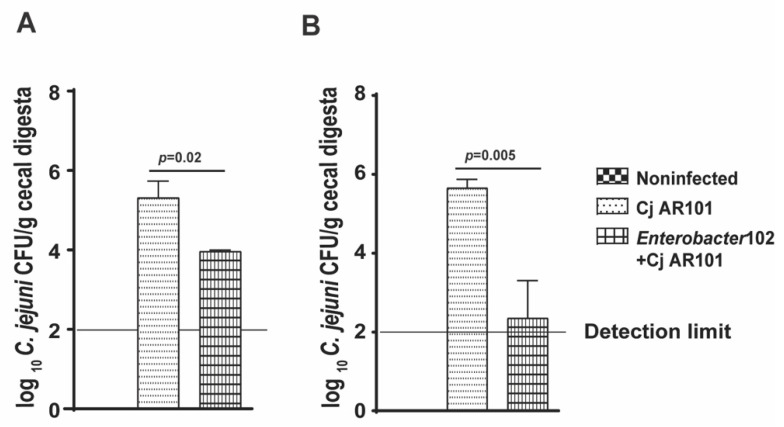
*Enterobacter*102-modulated *C. jejuni* AR101 chicken colonization. Zero-day-old broiler chicks were precolonized with *Enterobacter*102 and infected with *C. jejuni* AR101 at day 12. The birds were euthanized at days 21 and 28. The bird cecal digesta were collected, serially diluted, and cultured on *Campylobacter*-selective agar plates under a microaerobic atmosphere at 42 °C. (**A**) *C. jejuni* chicken colonization in the ceca of the birds at day 21. The bird number for each group was: noninfected (n = 10), Cj AR101 (n = 10), *Enterobacter*102 + Cj AR101 (n = 10). (**B**) *C. jejuni* chicken colonization in the ceca of the birds at day 28. The bird number for each group was: noninfected (n = 20), Cj AR101 (n = 20), *Enterobacter*102 + Cj AR101 (n = 20). All graphs depict the mean + SEM. Significant if *p* < 0.05. The results are representative of three independent experiments.

Encouraged by the result of *Enterobacter*102 reducing *C. jejuni* AR101 in vitro growth, we then performed chicken experiments. The birds were colonized with 10^8^ CFU/chick of *Enterobacter*102 at day 0, infected with *C. jejuni* at day 12, and euthanized at days 21 and 28. Consistently with the in vitro experiments, *Enterobacter*102 reduced *C. jejuni* AR101 chicken colonization by more than 1-log at day 21 in comparison to that in only infected birds of the Cj AR101 group (4.0 ± 0.0 vs. 5.3 ± 0.4 log_10_ *C. jejuni* CFU/g cecal digesta) (Figure 6A). Notably, *Enterobacter*102 continued to reduce *C. jejuni* AR101 chicken colonization by more than 2-log at day 28 (2.4 ± 0.9 vs. 5.7 ± 0.2 log_10_ *C. jejuni* CFU/g cecal digesta) (Figure 6B). These results suggest that the bacterial isolate of *Enterobacter*102 inhibited *C. jejuni* AR101 growth and reduced the pathogen’s chicken colonization.

## 3. Discussion

Poultry is the main reservoir of the prevalent foodborne bacterial pathogen *C. jejuni* which asymptomatically colonizes the birds [39]. However, the pathogen fails to colonize SPF or conventionally raised mice [30,38]. We then hypothesized that the microbiota from SPF mice might resist against *C. jejuni* infection, while the chicken microbiota might be susceptible to the pathogen. Here we report that mouse SPF-Aerobe and SPF-Anaero microbiota reduced *C. jejuni* chicken colonization at days 21 and 28. Notably, SPF-Aerobe and SPF-Anaerobe increased chicken cecal phylum *Bacteroidetes* and reduced phylum *Firmicutes* compared to those in the infected-alone birds. Interestingly, the uninfected chicken microbiota, SPF-Aerobe, or SPF-Anaerobe inhibited AR101 in vitro growth. Microbiota from birds transplanted with SPF-Aerobe or SPF-Anaerobe and infected inhibited AR101 in vitro growth, whereas microbiota from *C. jejuni*-infected-alone birds did not. *Enterobacter*102 isolated from infected birds transplanted with SPF-Aerobe reduced AR101 in vitro growth and chicken colonization. Altogether, these findings revealed that mouse SPF microbiota is able to colonize the chicken gut and resists against *C. jejuni* colonization in chickens, suggesting a potential strategy to reduce *C. jejuni* chicken contamination.

A notable observation from this study is that the mouse microbiota was able to be successfully transplanted into chickens and to reduce *C. jejuni* chicken colonization. It is a well-known medical practice to transplant a healthy donor’s microbiota to treat a human *Clostridium perfringens* infection [40]. The microbiota compositions of human recipients are comparable to those of the human donor’s, and the *C. difficile* infection is reduced. Consistently, microbiota composition in recipient piglets is similar to that of human donors in an intermammalian microbiota transplantation [41], suggesting that it is feasible to transplant microbiota between animals within the class level of Mammalia. In the current study, we successfully transplanted mouse (class Mammalia) microbiota to chickens (class Aves), suggesting it is possible to transplant microbiota between animals within the phylum level of Chordata. Apparently, the difference of body temperature (42 °C in chickens and 37 °C in mice) and intestinal anatomy between the animals did not reduce the donor mouse microbiota colonization in the recipient chickens. A meta-data analysis study showed that chicken microbiota at the phylum level is mainly comprised of 13 phyla, including *Firmicutes* (70.0%), *Bacteroidetes* (12.3%), *Proteobacteria* (9.3%), and other small proportions of *Actinobacteria*, *Cyanobacteria*, *Spirochaetes*, *Synergisteles*, *Fusobacteria*, *Tenericutes*, and *Verrucomicrobia* [42]. Consistent with this finding, we found that birds without a mouse microbiota transplantation had the phylum *Firmicutes* majority, while microbiota-transplanted birds dramatically reduced *Firmicutes* and increased *Bacteroidetes*, independently of *C. jejuni* infection. Interestingly, the microbiota in mice is composed of the phyla *Firmicutes* at 54% and *Bacteroidetes* at 30% [43], which is close to the composition of our transplanted chicken microbiota. A field survey study reported that birds from the farms with the highest *Campylobacter* counts show the highest percentage of *Firmicutes* and the lowest percentage of *Bacteroidetes* in their microbiota, although microbiota composition is highly variable between or within farms [44]. In addition, the significant reduction of *C. jejuni* colonization by SPF-Aerobe and SPF-Anaerobe microbiota in both days 21 and 28 suggested that the microbiota may continue to reduce pathogen colonization for a longer period of time. This experiment was cut short because of the pen size constrain. It would be interesting in the future to conduct follow-up experiments to reduce *C. jejuni* colonization by SPF microbiota for birds at the market age of days 35–45. Together, these data showed that mouse SPF microbiota is transplantable to reduce *C. jejuni* chicken colonization. 

After the evaluation of the protective effect of the mouse SPF microbiota, it is imperative to isolate and identify individual bacteria in the microbiota against *C. jejuni* chicken colonization for further functional evaluation. In a human longevity study, Sato and colleagues have plate-cultured, isolated, and evaluated a group of 68 bile acid metabolizing bacteria [45]. They found that *Parabacteroides merdae* and *Odoribacteraceae* strains produced isoalloLCA and reduced Gram-positive multidrug-resistant pathogens, such as *C. difficile* and vancomycin-resistant *Enterococcus faecium* [45]. A microbiota with higher level of genera *Clostridium XI*, *Bifidobacterium*, and *Lactobacillus* is associated with resistance to *C. jejuni*-induced colitis in mice [30]. Interestingly, probiotics *Bifidobacterium longum* PCB133 and a xylo-oligosaccharide do not decrease *C. jejuni* chicken colonization [46]. We have co-cultured *C. jejuni* with various ATCC or lab-isolated bacteria, such as *Bifidobacterium longum* and *Clostridium scindens*, and we did not find the bacteria to reduce *C. jejuni* in vitro growth (data not shown). During our search for individual microbiota against *C. jejuni*, we found that the *Enterobacter*102 from microbiota of SPF-Aerobe grew as pink colonies on the *Campylobacter*-selective plates. Later, we found that *Enterobacter*102 reduced *C. jejuni* in vitro growth and chicken colonization. Probiotic application of *Enterobacter* sp. improves both Mediterranean fruit fly (medfly) pupal and adult productivity and reduces rearing duration [47]. Most other reports showed that *Enterobacter* sp. is a pathogen and induces intesitnal inflammation [48,49]. Future research on how *Enterobacter*102 reduces *C. jejuni* growth and chicken colonization is much needed. We are working on identifying *Enterobacter*102 and other bacterial candidates by culture-isolation and 16S rDNA Sanger sequencing. Together, these data suggest that individual bacteria in the SPF microbiota might be able to be isolated and used to reduce *C. jejuni* growth and chicken colonization.

Another interesting finding from the current study is that the microbiota from noninfected birds at day 28 was able to reduce *C. jejuni* in vitro growth, while microbiota from infected-alone birds failed to reduce pathogen growth. The results suggest that *C. jejuni* might have modulated the chicken microbiota for facilitating pathogen colonizing and thriving in the gut. It is a consensus that intestinal microbiota influences *C. jejuni* colonization and induction of enteritis [24,50], as also discussed in the paragraphs above. However, few reports showed that *C. jejuni* modulates the microbiota to benefit its own colonization. *Salmonella* Enteritidis infection reduces the overall diversity of the chicken microbiota population with an expansion of the *Enterobacteriaceae* family for promoting pathogen colonization [51]. In the current study, we found that a *C. jejuni* infection increased the phylum *Bacteroidetes* compared to that in noninfected birds. Future research is needed to identify which specific bacteria are increased to facilitate *C. jejuni* colonization.

In conclusion, the mouse SPF microbiota was able to colonize chicken ceca and reduced *C. jejuni* chicken colonization. The reduction of *C. jejuni* chicken colonization might come from reduced bacteria in the phylum *Firmicutes* and/or increased bacteria in the phylum *Bacteroidetes*. Notably, *Enterobacter*102 reduced *C. jejuni* in vitro growth and chicken colonization. Altogether, these findings provide a feasible strategy to reduce *C. jejuni* chicken contamination and human campylobacteriosis.

## 4. Materials and Methods

### 4.1. Mouse Microbiota Preparation and Chicken Experiments of Microbiota Transplantation and C. jejuni Infection

The performed animal experiments were in accordance with the Animal Research: Reporting of In Vivo Experiments (https://www.nc3rs.org.uk/arrive-guidelines accessed on 22 August 2019) and approved by the Institutional Animal Care and Use Committee of the University of Arkansas (protocols No. 20009 for mice and 20011 for chickens). For the bird experiment with SPF microbiota, a total of 135 zero-day-old Cobb 500 broiler chicks were randomly allocated into cohorts of 15–30 birds per group, as detailed in Supplementary Table S1. The birds obtained from Cobb-Vantress (Siloam Springs, AR, USA) were neck-tagged and randomly assigned to floor pens with a controlled age-appropriate environment. The birds were fed a corn-soybean meal-based starter diet during days 0–10 and a grower diet during days 11–28. The basal diet was formulated as described earlier [37,52]. Stool from eight-week-old SPF BL6 *Il10*^−/−^ mice fed a chew diet was freshly collected and immediately suspended in 30% glycerol PBS stock and stored at −80 °C. The stool samples were cultured on brain heart infusion (BHI, BD Biosciences, Franklin Lakes, NJ, USA) agar plates at 42 °C for 48 h under aerobic or anaerobic conditions using the GasPak system (BD Biosciences, Franklin Lakes, NJ, USA) and collected as SPF-Aerobe and SPF-Anaerobe microbiota. The microbiota was added glycerol at final 30% and stored at −80 °C. Before the chicken colonization experiment, the SPF-Aerobe and SPF-Anaerobe microbiota were cultured on a BHI plate for 48 h, collected in PBS, and enumerated by OD_600_ and plating. OD_600_ of 1 was estimated at about 10^8^ CFU/mL. At chicken experiments, chicks at day 0 were orally gavaged once with PBS or 10^8^ CFU/bird SPF-Aerobe or 10^8^ CFU/bird SPF-Anaerobe. For the chicken experiment of *Enterobacter*102, a total of 90 zero-day-old Cobb 500 broiler chicks were randomly allocated into cohorts of 30 birds per group. The birds were fed and raised similarly to those in the SPF microbiota experiment. The chickens were orally gavaged once with PBS or 10^8^ CFU/bird of *Enterobacter*102 in 0.5 mL/bird on day 0. Two days before infection, frozen stock of *C. jejuni* AR101 (isolated at Dr. Billy Hargis’s lab at University of Arkansas at Fayetteville) were cultured microaerobically at 42 °C for 48 h on *C. jejuni*-selective blood plates. The motility of *C. jejuni* was ensured under a microscope as described before [53] and routinely examined on semisolid MH (0.4% agar) plates. *C. jejuni* AR101 in PBS was estimated as that OD_600_ of 0.468 was 10^10^ CFU/mL. The bacterium was also serially diluted, cultured on the *Campylobacter*-selective plates, and enumerated 48 h later. The *Campylobacter*-selective plate was prepared in-house and it consisted of Bolton’s Campylobacter Enrichment (CE) Broth (Neogen Food Safety, Lasing, MI, USA), 1.5% agar (VWR, USA, OH), 5% lysed horse blood (VWR, Radner Township, PA, USA), five antibiotics (20 mg/L cefoperazone, 50 mg/L cycloheximide, 20 gm/L trimethoprim, 20 mg/L vancomycin, and 0.35 mg/L polymyxin B), 500 mg/L ferrous sulfate, and 200 mg/L triphenyl-tetrazolium chloride (TTC) (all from Sigma-Aldrich, St. Louis, MO, USA). The ferrous sulfate and TTC were used to make *C. jejuni* colonies dark red. The birds were gavaged with 1 mL PBS or 10^9^ CFU/bird *C. jejuni* AR101 at day 12 [37]. Chicken body weight was measured at days 0 and 28. Because of the pen size constrains, the birds were randomly euthanized at days 21 and 28 to collect cecal samples for enumerating *C. jejuni*, and the exact bird numbers are listed in figure legends.

This experiment was conducted until 28 days of age because of the pen size constrain. Cecal digesta samples of all the groups were collected for DNA isolation. Another set of cecal digesta were serially diluted ten-fold with sterile PBS and cultured on the *Campylobacter*-selective plates at 42 °C for 48 h under a microaerophilic atmosphere. Emerged colonies were positively determined as *C. jejuni* only when they were dark red and shining, round, and with a smooth surface. The colonies were also examined under a microscope for size and motility evaluation [53]. The CFU per gram digesta was then calculated.

### 4.2. Estimation of Microbiota Composition at Phylum Level

Cecal digesta samples were collected, and DNA was extracted using bead beater disruption and phenol: chloroform separation method as described before [54]. Briefly, 0.1 g of fecal sample suspended in 500 μL PBS was transferred to a 2 mL screw cap tube containing 85 μL of 10% SDS solution, 500 μL of phenol/chloroform (25:24), and 0.3 g sterile 0.1 mm zirconia beads (BioSpec, Bartlesville, OK, USA). The samples were homogenized on a Fisher brand Bead Mill 24 Homogenizer (Fisher Scientific, Pittsburg, PA, USA) for 3 × 30 s at high speed with a 10 s pause for each run. After centrifugation, the supernatant was further extracted twice with 500 μL of chloroform (25:24), and the top aqueous layer was collected and mixed with 1/10 Vol (~50 μL) 3M sodium acetate (pH 5.2) and 2.5 Vol (~1.25 mL) ethanol overnight at –20 °C. After centrifugation, the DNA pellet was washed once with 70% ethanol and resuspended in 100 μL DNase/RNase-free H_2_O. The abundance levels of five phyla of gut bacteria were determined by real-time PCR according to the manufacturer’s recommendation. Briefly, each PCR reaction mixture comprised 4 μL of BioRad iTaq Universal SYBER Green Super mix (BioRad, Hercules, CA, USA), 1.6 μL of template DNA (~4 ng), 0.6 μL of 5 μM primer mix, and 1.8 μL of DNase/RNase H_2_O. The amplification reaction was performed in a BioRad 384 Real-Time PCR machine (BioRad, Hercules, CA, USA) using the following program: 1 min at 95 °C, followed by 30 cycles of 30 s each at 95 °C, 60 s at 60 °C. The gene primers [37] used included universal 16S rRNA: 16S357F 5′-CTCCTACGGGGAGGCAGCAA-3′, 16S1392R 5′-ACGGGCGGTGTGTRC-3′; *α-proteobacteria*: α682F 5′-CIAGTGTAGAGGTGAAATT-3′, 908αR 5′-CCCCGTCAATTCCTTTGAGTT-3′; *γ-proteobacteria*: 1080γF 5′-TCGTCAGCTCGTGTYGTGA-3′, γ1202R 5′-CGTAAGGGCCATGATG-3′; *Bacteroidetes*: 798cfbF 5′-CRAACAGGATTAGATACCCT-3′,cfb967R 5′-GGTAAGGTTCCTCGCGTAT-3′; *Firmicutes*: 928FirmF 5′-TGAAACTYAAAGGAATTGACG-3′, 1040FirmR 5′-ACCATGCACCACCTGTC-3′; *Actinobacteria*: Act920F3 5′-TACGGCCGCAAGGCTA-3′, Act1200R 5′-TCRTCCCCACCTTCCTCCG-3′. The relative percentage of each phylum was calculated following the relative PCR quantification method [55] similar to that in this paper [55]. Briefly, the 2^−ΔΔCT^ value of each phylum gene expression Ct in one sample was calculated using the universal 16S rRNA gene expression Ct. The percentage of each phylum was then calculated by the phylum 2^−ΔΔCT^ value in one sample divided by the sum of all phylum 2^−ΔΔCT^ values in the same sample and multiplied by 100.

### 4.3. Isolation of Enterobacter102

When chicken cecal digesta were cultured on *C. jejuni* selective plates, pink colonies were grown on the plate, compared to dark red *C. jejuni* colonies. The pink colony was named *Enterobacter*102. Under a light microscope, *Enterobacter*102 was rod-shaped and larger than *C. jejuni*. *Enterobacter*102 was able to grow aerobically, stained Gram-negative, and showed pink colonies on a MacConkey plate.

### 4.4. Identification of Bacterial Species Using 16s rDNA and Sanger Sequencing

Either *C. jejuni* AR101 or *Enterobacter*102 was derived from a single colony. To isolate DNA for Sanger sequencing, the bacteria were spread on the respective agar plates. The bacteria were collected, and DNA was extracted. Genomic DNA from *C. jejuni* AR101 or *Enterobacter*102 was amplified by PCR of the 16S rDNA gene region with universal primers (27Fw1: 5′-AGAGTTTGATCMTGGCTCAG-3′, 1492R: 5′- CGGTTACCTTGTTACGACTT-3′) following the instructions in this webpage https://chmi-sops.github.io/mydoc_16S_Sanger.html (accessed on 20 July 2020). The PCR products were gel-purified and Sanger-sequenced at Eurofins Scientific using primers of 27Fw1, 1492R, and universal primer 515Fw2: 5′-GTGCCAGCMGCCGCGGTAA-3′. The sequences were assembled and aligned using the NCBI genome database. The bacteria were given species names with >98.0% and 95.0% of 16S rDNA sequence homology for *Campylobacter jejuni* and *Enterobacter* sp*.,* respectively. The 16s rDNA sequences were uploaded at NCBI with submission numbers of SUB10285129 and SUB10285090.

### 4.5. In Vitro Co-Culturing of C. jejuni with Various Microbiota

The impact of various microbiota on *C. jejuni* growth was evaluated. Briefly, *C. jejuni* AR101 from frozen stocks was cultured and grown on the *Campylobacter*-selective plates in a microaerophilic atmosphere for 48 h using the GasPak system (BD Biosciences, Franklin Lakes, NJ, USA). *C. jejuni* at 6.3 × 10^8^ CFU was co-cultured with noninfected microbiota at 2.0 × 10^8^ CFU, SPF-Aerobe at 1.8 × 10^8^ CFU, or SPF-Anaerobe at 8.4 × 10^8^ CFU in 1 mL of CE broth. *C. jejuni* at 7.7 × 10^8^ CFU was co-cultured with Cj-MB at 6.7 × 10^7^ CFU, Cj-SPF-Aerobe at 1.6 × 10^9^ CFU, or Cj-SPF-Anaerobe at 1.5 × 10^9^ CFU in 1 mL of CE broth. *C. jejuni* at 1.8 × 10^8^ CFU and 4.4 × 10^7^ CFU *Enterobacter*102 were co-cultured in 1 mL of CE broth. The experiments were carried out in triplicate. Because *C. jejuni* growth would be reduced within 24 h in anaerobic conditions [54], the co-culture bacteria were incubated for 24 h at 42 °C under anaerobic conditions using the GasPak system (BD Biosciences, Franklin Lakes, NJ, USA) to mimic cecal air conditions. *C. jejuni* growth was measured by serial dilution and plating on the *Campylobacter*-selective plates for enumeration. When co-culture with *C. jejuni*, *Enterobacter*102 was counted with pink colonies compared to dark red colonies of *C. jejuni*. Cj-MB, Cj-SPF-Aerobe, and Cj-SPF-Anaerobe themselves could not grow a single colony on the *Campylobacter*-selective plates, suggesting that *C. jejuni* lost culturability after storing with microbiota.

### 4.6. Statistical Analysis

All values are shown as mean ± standard error of the mean as indicated. Differences between groups were analyzed using the nonparametric Mann–Whitney U test performed using GraphPad Prism 7.0 software. *C. jejuni* CFU was transformed with a formula of log10 (CFU + 1). The results were considered statistically significant if *p*-values were <0.05.

## Figures and Tables

**Table 1:** Significant *p*-values of relative abundance at the phylum level between groups.

Group A	Compared to Group B	Phylum	*p*-Value
Noninfected	SPF-Aerobe	*Bacteroidetes*	<0.001
		*Firmicutes*	<0.001
	SPF-Anaerobe	*Bacteroidetes*	<0.001
		*Firmicutes*	<0.001
	Cj AR101	*Bacteroidetes*	0.02
		*Firmicutes*	0.04
Cj AR101	SPF-Aerobe + Cj AR101	*Bacteroidetes*	<0.001
		*Firmicutes*	<0.001
	SPF-Anaerobe + Cj AR101	*Bacteroidetes*	<0.001
		*Firmicutes*	<0.001

## Data Availability

The data are presented in this paper.

## Supplementary Materials

The following are available online at https://www.mdpi.com/article/10.3390/pathogens10111387/s1, Figure S1. Accumulative body weight gain during days 0–28. Figure S2. SPF microbiota growth co-cultured with *C. jejuni* for 24 h. Figure S3. Cj-SPF microbiota growth co-cultured with *C. jejuni* for 24 h. Figure S4. *Enterobacter*102 growth co-cultured with *C. jejuni* for 24 h. Table S1. Number of birds in each group of SPF microbiota experiments.
