# Supplementary material for: Microbiota from Specific Pathogen-Free Mice Reduces Campylobacter jejuni Chicken Colonization
Source: Pathogens. 2021 Oct 27;10(11):1387. doi: 10.3390/pathogens10111387 (PMC8621964; doi:10.3390/pathogens10111387)
Supplement: Supplementary file 1 [file pathogens-10-01387-s001.zip › pathogens-1381723-supplementary.pdf]

pathogens

MDPI

Supplementary materials

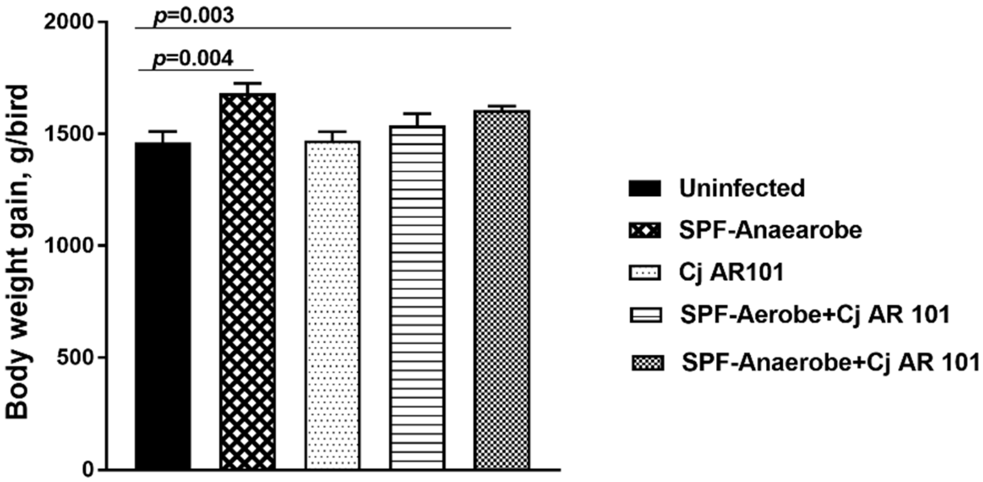

Figure S1. Accumulative body weight gain during d 0-28. Cohorts of chicks were colonized with mouse SPF microbiota and infected as in Figure 1. The bird weight was measured at d 0 and 28. The bird number was same as d 28 in Figure 1. Showed were accumulative body weight gain during d 0-28. All graphs depict mean + SEM. Significant if $p < 0.05$. Results are representative of 3 independent experiments.

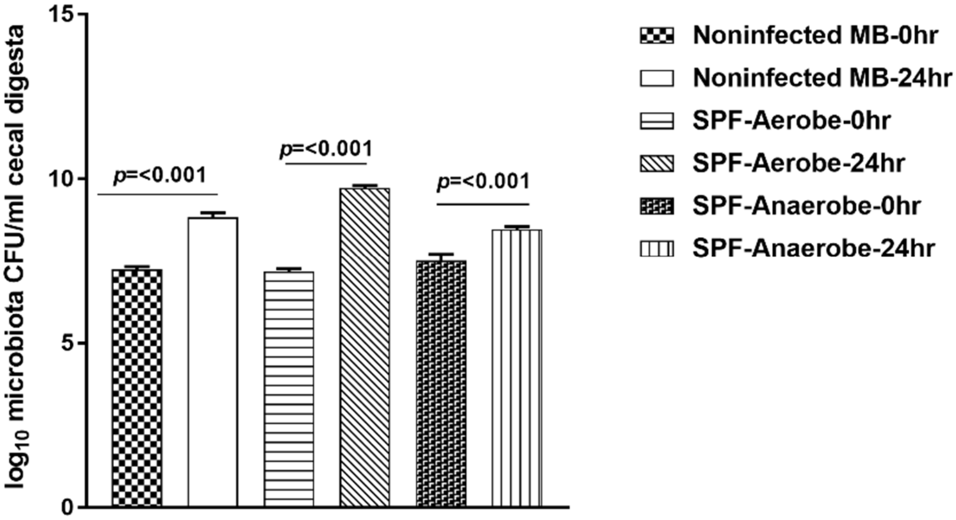

Figure S2. SPF-Microbiota growth co-cultured with *C. jejuni* for 24 hr. SPF microbiota was co-cultured with *C. jejuni* for 24 hr as described in Figure 3. Microbiota growth was quantified by serially diluting and plating on BHI plates. All graphs depict mean + SEM. Significant if $p < 0.05$. Results are representative of 3 independent experiments

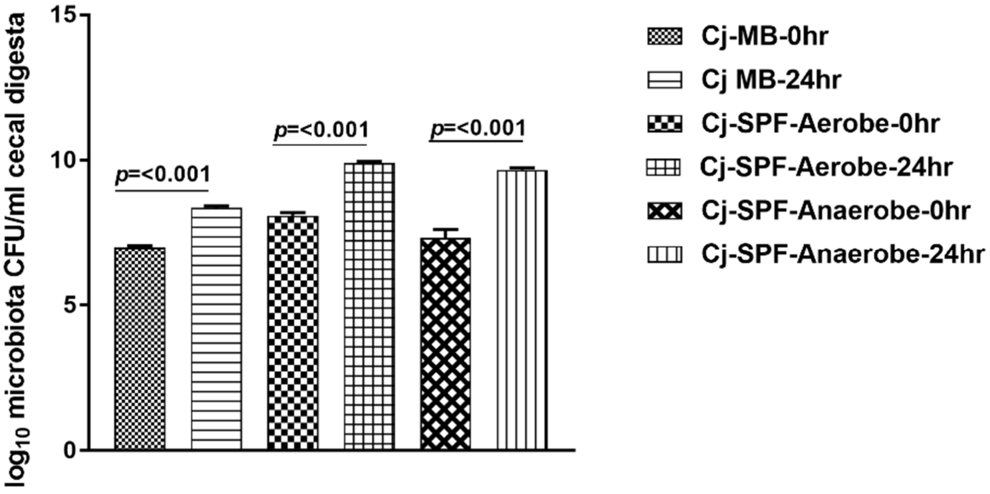

Figure S3. Cj-SPF-Microbiota growth co-cultured with *C. jejuni* for 24 hr. Cj-SPF microbiota was co-cultured with *C. jejuni* for 24 hr as described in Figure 4. Microbiota growth was quantified by serially diluting and plating on BHI plates. All graphs depict mean + SEM. Significant if *p* <0.05. Results are representative of 3 independent experiments.

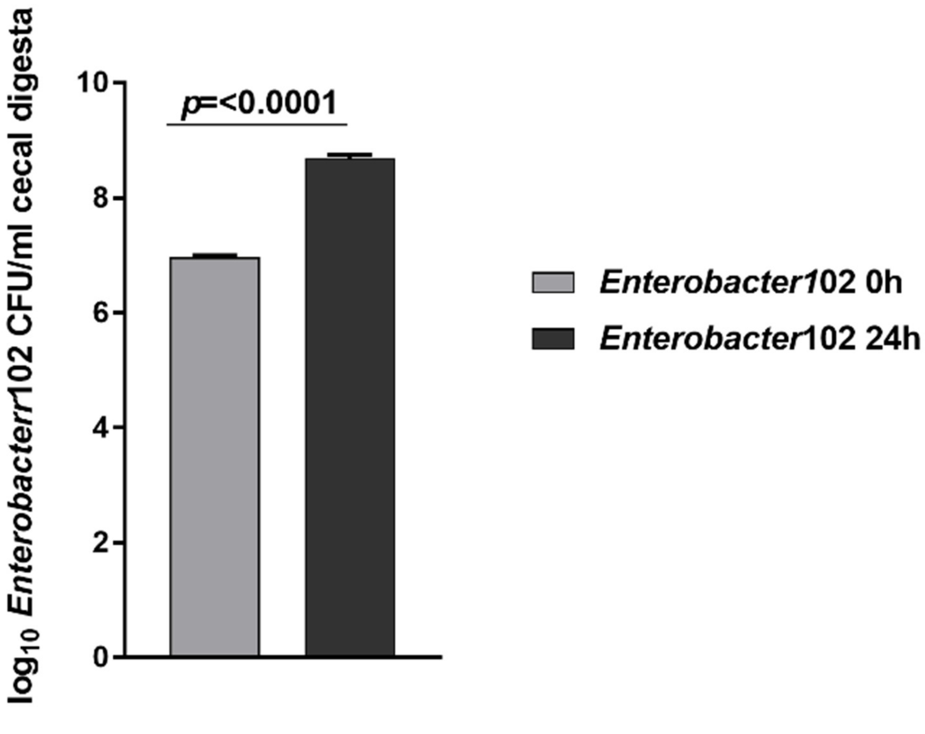

Figure S4. *Enterobacter*102 growth co-cultured with *C. jejuni* for 24 hr. *Enterobacter*102 was co-cultured with *C. jejuni* AR101 for 24 hr. *Enterobacter*102 growth was quantified by serially diluting and plating on BHI plates. All graphs depict mean + SEM. Significant if *p* <0.05. Results are representative of 3 independent experiments.

Supplementary Table S1. Number of birds in each group of SPF microbiota experiments.

Group	Pens/group	Number of birds/group
Noninfected	2	30
SPF-Aerobe	2	30
Cj AR101	2	30
SPF-Aerobe+ Cj AR101	1	15
SPF-Anaerobe+ Cj AR102	2	30
